# CurlySMILES: a chemical language to customize and annotate encodings of molecular and nanodevice structures

**DOI:** 10.1186/1758-2946-3-1

**Published:** 2011-01-07

**Authors:** Axel Drefahl

**Affiliations:** 1 Axeleratio, 4330 Tuscany Circle, Reno, Nevada 89523, USA

## Abstract

CurlySMILES is a chemical line notation which extends SMILES with annotations for storage, retrieval and modeling of interlinked, coordinated, assembled and adsorbed molecules in supramolecular structures and nanodevices. Annotations are enclosed in curly braces and anchored to an atomic node or at the end of the molecular graph depending on the annotation type. CurlySMILES includes predefined annotations for stereogenicity, electron delocalization charges, extra-molecular interactions and connectivity, surface attachment, solutions, and crystal structures and allows extensions for domain-specific annotations. CurlySMILES provides a shorthand format to encode molecules with repetitive substructural parts or motifs such as monomer units in macromolecules and amino acids in peptide chains. CurlySMILES further accommodates special formats for non-molecular materials that are commonly denoted by composition of atoms or substructures rather than complete atom connectivity.

## Background

CurlySMILES (Curly-braces enhanced Smart Material Input Line Entry Specification) is introduced as a chemical language for the specification of chemical materials and supramolecular structures. The CurlySMILES approach provides a flexible format to encode patterns in materials and molecule-based architectures. CurlySMILES includes its own set of symbols, descriptors and rules to denote respective entities and also modifies the well-established SMILES language.

SMILES is based on a set of rules that allow the representation of a molecular structure as a sequence of atom and bond symbols in a single word or string [1-3]. Unique SMILES strings are suitable as database keys while storing the structural information within the key itself [4]. Since SMILES notations are constructable from molecular principle, namely the molecular graph, notations can be derived for virtual, not-yet-synthesized chemical species. The flexibility and portability of SMILES has been demonstrated by its use in modeling and property estimation software [5-9] and combinatorial libraries [10,11].

SMILES is bridging the opposite ends of human-friendly molecular drawings, achieved with molecule editors [12,13], and computer-friendly connection tables (matrices), both used in representing molecular structures. Molecular information collapsed into a compact SMILES string can efficiently be managed by computer programs and stored in markup language fields, like the <sSmiles> tag of ThermoML [14].

SMILES comes in various dialects with modifications or minor extensions of the originally published language. Implementations of SMILES parsers differ with respect to the treatment and acceptance of additionally introduced symbols and syntax [2]. SMILES also has been extended to encode a peptide or peptoid sequence on monomer level [15] and in template format [16].

Recently, the IUPAC International Chemical Identifier (InChI) has been designed as a string-based identifier for chemical substances [17]. Like a SMILES notation, an InChI string is derived from a molecular structure representations. However, InChI is intended for "behind-the scenes" use by computers. It is typically derived from structure representations by software, whereas SMILES handily supports molecular communication between humans and computers.

The user-friendliness and popularity of SMILES encouraged us to modify this chemical language for communication of molecular architectures that can not adequately be encoded with the current SMILES language and its derivatives. CurlySMILES modifies the SMILES language by including a novel format to encode molecular details and extra-molecular features such as non-covalent interactions and attachment to a biomolecule as well as the surface of a substrate material or nanoparticle. CurlySMILES is designed with an open format to provide users with choices of integrating shorthands such as aliases or compaction of repetitive structural units. In the following, formats and rules for constructing CurlySMILES notations are described. Applications of CurlySMILES for document annotation and chemical search are then discussed.

## Results

### CurlySMILES notation

A CurlySmiles notation is a string of dot-separated component notations. A component notation can either be a plain SMILES, an annotated SMILES, or a special format notation. A plain notation maintains the grammar and rules of the known SMILES language. A plain notation is modified by introducing attributes, such as structural variations, details and decorations, enclosed in curly braces. An annotation can be anchored to a particular atomic node or placed at the end of a SMILES component. A special format notation begins with an opening and ends with a closing curly brace and includes an alias or a notation for a structure that defies molecular-graph encoding. A string with exactly one component notation is referred to as a unary CurlySMILES notation.

A CurlySMILES notation is typically not unique since the SMILES language allows for alternate notations by selecting a starting atom arbitrarily. Further, CurlySMILES provides flexible annotations formats that leaves it to a user or application software to add and granulate details. The clear separation of attributes from the molecular-graph encoding, however, provides applications with options to match and screen notations in large data sets with precedence to attributes, while deferring molecular-connectivity processing to a later stage, at which only a selected set of candidates will be considered.

Here, we focus on the core format of CurlySMILES, outlining the basic syntax that can be extended into different domains of future interest. Example notations are supplied for selected molecules and materials, demonstrating how to represent a targeted structure or a generically defined class of structures. More examples are available in Additional file 1 and on the Web [18,19].

Example notations are displayed in monospace font. Parts of a CurlySMILES notation, which are given in *italics*, present descriptive metalanguage text meant to be replaced by code in CurlySMILES format.

### Multiplier

A shorthand format is introduced to encode multiple occurrences of the same component notation. The multiplier is an integer greater than one and enclosed in curly braces. It is appended to a component notation as illustrated for cobalt(II) nitrate hexahydrate (Co(NO_3_)_2_·6H_2_O):

[Co+2].[O-]N(=O)=O{2}.O{6}   multiplier notation,

[Co+2].[O-]N(=O)=O.[O-]N(=O)=O.O.O.O.O.O.O   exhaustive notation.

A multiplier is not considered an annotation. If annotations occur at the end of a component notation, they have to precede the multiplier.

### Alias

An alias is a short form for a component notation. An alias is enclosed in curly braces. CurlySMILES distinguishes between predefined and customer-defined aliases.

This distinction is critical for the implementation of a CurlySMILES parser. Look-up of the replacement notation for an alias is internal for a predefined alias, whereas customer-defined aliases require submission of a look-up dictionary by the customer.

An alias begins with a letter or a dollar sign followed by zero or more alphanumerical, hyphen, underscore, plus sign and round bracket characters. For example, we use commonly applied short notations for cations and anions of ionic liquids and solids, such as bmim and NTf2 for 1-butyl-3-methylimidazolium and bis[(trifluoromethyl)sulfonyl]imide, respectively. This allows the following shorthand encoding:

{bmim(1+)}.{NTf2(1-)}   1-butyl-3-methylimidazolium bis[(trifluoromethyl)sulfonyl]imide.

A customer-defined alias has to be indicated by a preceding dollar sign, allowing for notations like the following:

{$*myCation*}.{$*myAnion*}.{$*mySolvate*}{4}   aliases-customized notation.

### Stoichiometric formula notation

A stoichiometric formula notation (SFN) is defined to encode materials with known atomic or substructural stoichiometry, but without a discrete pattern of finite atom connectivity (molecular structure) that could be captured in a SMILES notation. SFNs are particularly useful for encoding a broad range of solids. Further, many homo- and hetero-polyatomic clusters with complex atom connectivity can be encoded as SFNs in a compact, yet distinctive manner. CurlySMILES applies an SFN format that resembles the nomenclature typically used to name compounds by their stoichiometric composition [20], but eliminates the use of sub- and superscript markup. Multiple entries of the same atomic symbol are allowed and the symbols may occur in any order. A stoichiometric integer directly follows a symbol, whereas an isotope label precedes a symbol and is marked with the ^ character; for example ^13C, for ^13^C. Further, selected atomic symbols may be grouped by enclosing them within round braces. If a stoichiometric integer applies to a group, it immediately follows the closing round brace. Finally, a charge notation (n+) or (n-), where n≥1, is placed at the end of a SFN notation for ionic species.

To distinguish an SFN-encoded component from an alias or a composite notation, an asterisk (*) precedes the SFN. The general format is {*SFN}. A few examples illustrate SFN encoding in CurlySMILES:

{*Cr23C6}   tricosachromium hexacarbide, Cr_23_C_6_

{*Bi5(4+)}   pentabismuth(4+) cation, Bi_5_^4+^

{*Cu3(CO3)2(OH)2}   azurite, Cu_3_(CO_3_)_2_(OH)_2 _(carbonate mineral)

Groups in an SFN notation can be nested to any depth level:

{*K(AuS(S2))}   potassium (disulfido)sulfidoaurate(1-), K[AuS(S_2_)]

Notice that the SFN format also accommodates structures with molecular connectivity. It is the user's choice to encode such structures as SFN or SMILES notations. When in doubt about the topological description of a structure or when isomeric forms should intentionally be included, SFN is the notation of choice.

An SFN can appear within a special format notation for a component, within a composite notation to encode a constituent (see next section) and within a SMILES annotation.

### Composite notation

Composite or hybrid materials are made from constituents that remain separate and distinct, even on an above-nanoscale level (mesoscale). The constituents should not be encoded by dot-separated notations, a format which should be reserved for compounds in which the different species interact with each other on a molecular/ionic level rather than on an interface or grain-boundary level. CurlySMILES defines a special format for encoding composites and other materials built from interface-connected phases:

{/*constituent*#1/*constituent*#2/.../*constituent*#n}

Herein, *constituent*#i is the constituent notation of the i-th constituent, which is either an SFN or a SMILES component notation. The forward slash between two adjacent constituents, which is typically used in the scientific literature for materials and device structures of this type, symbolizes a contact through a common interface. A composite notation is always a unary CurlySMILES notation. For example, a composite material based on poly[bis(methoxy-ethoxy-ethoxy)phosphazene] (MEEP) and zirconium dioxide [21] is encoded as

{/N{-}=P{+n}(OCCOCCOC)(OCCOCCOC)/{*ZrO2}}   MEEP/ZrO_2_

The first constituent is presented as an annotated SMILES notations, using the macromolecule syntax introduced below, and the second constituent is presented as SFN.

### Plain SMILES notation

A component notation that does not begin with an opening curly brace is a plain SMILES or an annotated SMILES notation. A plain SMILES notation is encoded with the grammar and rules of the SMILES language [1]. A plain notation contains atomic node code (ANC) and may contain bond symbols and special characters to denote branching and ring formation. An ANC is either a bare atomic symbol or a square bracket atomic code (SQC) which includes an atomic symbol and, depending on the targeted structure, additional characters to denote the number of adjacent hydrogen atoms, a charge value, and an isotope label. CurlySMILES includes the symbols *, ~, and $ to encode an atomic wildcard, an unspecified bond and a quadruple bond, respectively. Stereodescriptive symbols such as @, /and \ are not accepted, since CurlySMILES provides a special annotation syntax to specify and distinguish stereoisomers.

CurlySMILES requires an atomic wildcard always to be encoded as a SQC. For example, the notation [*H2]=[*] encodes a molecule in which one atom with two adjacent hydrogen atoms is connected by a double bond to another atom, which has no further neighbor atoms. The symbol ~ was introduced in SMARTS [22] as a wildcard-like any-bond symbol. Unless the bound atoms are encoded by wildcards, a bond is predetermined by the element type of the adjacent atoms and their orbital interactions. Whereas the atomic wildcard is a placeholder for atomic symbols, the symbol for an unspecified bond has not primarily the role of a bond placeholder; rather, it indicates bond-type ambiguity with respect to the limited classification scheme of single, double, triple, quadruple and aromatic bonds. CurlySMILES treats the character ~ as a bond symbol that encodes a bond, which cannot adequately be encoded as a covalent bond with symbols -, =, #, :, and $. The latter symbol is used to encode a quadruple bond. CurlySMILES requires all atomic symbols, that encode atoms connected by a $ or ~ bond, to be encoded in SQC notation. As described below, CurlySMILES provides an annotation format that allows encoding of bond details.

### Annotated SMILES notation

A plain notation is annotated by inserting one or more curly-enclosed annotations into the notation. An annotation has to be either anchored at a particular atomic node or appended to the end. An atom-anchored annotation (AAA) directly follows the ANC. The only characters allowed to occur between an AAA and ANC are digits that designate ring-closing. A component-anchored annotation (CAA) follows the last ANC, including its AAAs, but precedes the multiplier, if any is present in the component notation. AAA types include stereodescriptive and structural unit annotations as well as group environment, molecular detail and operational annotations. CAA types include state and shape annotations and miscellaneous interest annotations. In the following text we use the term annotation to refer to the content between the curly braces.

A stereodescriptive annotation consists of one of the upper-case letters D, E, L, R, S, and Z. There meaning corresponds to their definition in the chemical nomenclature: R and S mark a stereogenic atom, D and L mark an atom to relate a chiral molecule to an enantiomer of glyceraldehyde, and E and Z distinguish *cis*/*trans *isomers by using the *E*/*Z *convention. Examples E1 and E2 in Additional file 1 demonstrate stereodescriptive annotation.

A structural unit annotation defines a boundary of a structural unit. This boundary is an open or dangling bond. The annotation consists of a one-character boundary descriptor, which is a CurlySMILES bond symbol (-, =, #,:, &, or ~). For descriptors:, &, and ~, the anchor atom always has to be SQC-encoded. For -, =, and #, SQC-encoding is not required as long as the anchor atom belongs to the organic set and has no isotope label or charge contribution. Structural unit annotations apply to the encoding of formal fragments or groups. Their use is demonstrated in E3 in Additional file 1.

Stereodescriptive and structural unit annotations consist of exactly one character, while all other annotation types require a two-character annotation marker (AM), which is optionally followed by an annotation dictionary to specify attributes. The general format for a dictionary-containing annotation,

{*AMk*_1_=*v*_1_;*k*_2_=*v*_2_;...;*k*_n_=*v*_n_},

employs a semicolon-separated list of dictionary entries. An entry is a key/value pair, *k*_i_/*v*_i_. A key is a short name which starts with a letter or a dollar sign followed by zero or more letters or underscore characters. A beginning dollar sign indicates a customized entry, in which key name and possible values have to be defined by the user. Otherwise, key names and associated values are predefined [23].

A group environment annotation marker (GEAM) starts with a bond symbol (-, =, #, :, &, ~) or a dot. A bond symbol followed by Y describes bonding to an adjacent substructure. Y is to be interpreted generically as any substructure, but can be specified through dictionary entries. The bond symbol - may alternately be followed by R or X to indicate an alkyl or halogen group. Symbols - and ~ can be followed by a vertical bar to indicate surface attachment. Further, the GEAM. | indicates ionic interaction with a surface. Examples are given below and in E4 and E5 in Additional file 1.

A molecular detail annotation starts with an exclamation mark. The second character of a molecular detail annotation marker (MDAM) is a, p, m, r, H, and I denoting anchor atom, pair of atoms, multiplet of atoms, ring, hydrogen-bonding and non-hydrogen-based interaction, respectively. Examples E6 to E8 in Additional file 1 illustrate their use.

The first character of an operational annotation marker (OPAM) is a plus sign. The following character is a letter. An upper-case letter indicates formal addition or substitution of a structural part. A lower-case letter indicated formal repetition of an annotated unit. OPAMs +R, +X, and +Y mean formal substitution of a H-atom by an alkyl group, halogen atom and "any group", respectively. OPAM +L means ligand addition and is used to encode coordination compounds. OPAMs +n and +r formally encode linear marcomolecules and macrocycles, respectively. Examples E9 to E14 in Additional file 1 provide details on their use.

A state and shape annotation is denoted by a state and shape annotation marker (SSAM) consisting of two lower-case letters. SSAM annotations qualitatively describe the physicochemical state, phase structure and/or the nano- or mesoscale characteristics of a material [24]. Examples E15 to E18 in Additional file 1 show their application.

A miscellaneous interest annotation is denoted by a two-character miscellaneous annotation marker (MIAM). Examples E19 and E20 in Additional file 1 illustrate their employment.

The following two examples demonstrate the combined use of annotations to encode molecular arrangements.

**Figure 1:**
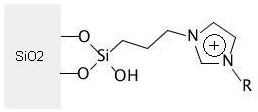
**Alkylimidazolium ionic species immobilized on silica surface**.

The imidazolium functionalized SiO_2 _surface [25] in Figure 1 is encoded in CurlySMILES by applying the group environment annotation format. The two O atoms, which attach the molecular species to the material surface, are annotated with the -|marker:

O{-|sfn=SiO2}[Si](O{-|i=-2})(O)CCCn1ccn{-R}c1{!re=+}   (Figure 1)

The material is SFN-encoded in a dictionary entry with key sfn. Instead of repeating this entry in the annotation of the second surface-attached O-atom, that annotation contains an entry with key i, pointing to the atomic node with the material-surface description. The value -2 means that one has to move to the left by two atomic nodes to locate the relevant node. This notation further contains annotation {-R} at the N atom of the imidazolium ring to specify an alkyl substituent. The delocalization of the ring charge is encoded by annotating the node at which the ring is formally closed. The molecular detail annotation, marked with !r, includes the dictionary entry e=+, which denotes the positive ring charge.

**Figure 2:**
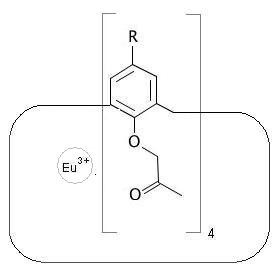
**Eu^3+ ^cation coordinated by a cryptand**.

The functionalized calix[4]arene of the complex shown in Figure 2 is encoded by combining structural unit, group environment and operational annotations. The molecular ring is encoded as a fragment with two open bonds. The first is encoded as a structural unit annotation and the second as an operational annotation. Corresponding atomic nodes and annotations are given in boldface:

CC(=O)COc1**c{-}**cc{-R}cc1**C{+rn=4}**   functionalized calix[4]arene

The +r annotation formally instructs to close the open single bond at the C atom of the last structural unit with the open single-bond at the aromatic-ring C atom of the first structural unit after repeating the unit as a ring constituent four times. The complete Eu^3+^/cryptand complex is given by encoding the rare-earth cation in SQC, annotated with the ligand notation:

[Eu+3]{+Lc=CC(=O)COc1c{-}cc{-R}cc1C{+rn=4}}   (Figure 2)

The dictionary key c expects a CurlySMILES notation. This example demonstrates that CurlySMILES notation can be recursive. Occurrence of notations at different hierarchical levels allow selective and role-specific parsing and interpretation of CurlySMILES notations within application context.

### Software

A suite of Python modules have been implemented that perform parsing and molecular descriptor generation for CurlySMILES notations. These modules have been wrapped as software package csmpy-1.0.1.tar.gz, which is released under the terms of the GNU General Public License (GPLv3) as published by the Free Software Foundation. The package can be downloaded from http://www.axeleratio.com/csm/py/code/downloads.htm.

## Discussion

### Comparison of CurlySMILES with other SMILES modifications

Versions of SMILES use the symbols @ and @@ to distinguish an asymmetric atom in a chiral molecule. *Cis *and *trans *isomers are denoted via directional bond symbols / and \ around a double bond. In CurlySMILES, corresponding notations for stereoisomers are encoded by inserting annotations containing R and S (optionally D and L for certain configurations) and E and Z, depending on the actual configuration. Stereodescriptor assignment in CurlySMILES follows the rules of chemical nomenclature. This allows for a direct correspondance between descriptors in a systematic name and an associated CurlySMILES notation.

CurlySMILES introduces a format to mark a fragment or chemical group by using structural unit annotations. This allows distinction between a radical and a group. The SMILES notation [CH3] encodes a methyl radical. CurlySMILES uses the same notation to encode the radical, but encodes a methyl group as C{-}.

In a CurlySMILES notation, SMILES code is strictly separated and distinguishable from other parts in the notation by using curly braces. CurlySMILES uses marked annotations to define structures in generic terms or to construct molecular patterns with a tunable depth of information granularity. CurlySMILES provides completely new, extra-molecular patterns, such as [*]{+Rn=4-16}{-|}, which reads as "branched or unbranched alkyl group with four to sixteen C-atoms at any bivalent hydrogen-free atom that is grafted to a material surface by a single bond." The possibility of encoding surface-attached molecules enhances the use of the SMILES language for encoding and querying nanostructures and molecular devices, which often consists of molecules attached to the surface of quantum dots, nanowires, nanotubes and other nano-objects. Those architectures often include non-molecular solids such as semiconductors, alloys, and ceramics. The SFN format of CurlySMILES provides a formalism to encode these materials on their own. The SFN notation also is critical in encoding certain composite phases or in specifying matrix materials or solid interfaces. Combinatorial libraries of functionalized surfaces and nanoparticles can efficiently be generated and/or screened by applying these versatile encoding options.

CurlySMILES is designed for applications in specialized domains and for clients with particular tasks, including repetitive processing of certain structural entities. For this purpose, CurlySMILES includes various shorthand approaches, especially the alias format. Domain-specific abbreviations and codes for structures and materials are frequently used within chemical communities to replace long names and complex structural concepts. The alias format makes it possible to integrate those terms into notations and replace them when needed.

As a machine-readable code, a CurlySMILES notation (like SMILES or InChI) is a document-neutral representation (ASCII string) of a chemical structure that, supported by the methods in the supplied software package, can automatically be converted into a document specific format. Formula-based names of coordination compounds are a case in point.

### Data mining and semantic search

A special feature of CurlySMILES is that it integrates textual parts, acronyms, other encoding schemes and client-defined aliases. Thus, a CurlySMILES notation can be used on various levels in search and data mining. CurlySMILES allows formulation of complex search pattern by using atomic-symbol placeholders and annotations that denote generic functional groups and compound classes. The annotation syntax of CurlySMILES makes it possible to associate a structural part with a specific role such as a structural repeat unit in a polymer, a substituent, ligand, cryptand, dopant, adsorbate or dissolved species. By implementing search and matching algorithm that include the information contained in CurlySMILES annotations, a plethora of search strategies can be envisioned, including precise, needle-in-the-hay-stack search and customer-focused report-and-review style extraction of chemical data.

## Conclusions

CurlySMILES is a chemical language for the communication of chemical information related to molecular structure and complex nanoscale architectures. This language offers a versatile approach in encoding material composition and structure by supplementing attention to extra-molecular features. Symbols and grammar of this language allow users to encode structures and to formulate context-annotated queries with variable granularity of molecular or supramolecular details. The open format makes it easy to extend the current version to application-specific tasks.

## Abbreviations

AAA: Atom-Anchored Annotation; AM: Annotation Marker; ANC: Atomic Node Code; CAA: Component-Anchored Annotation; CurlySMILES: Curly-braces enhanced Smart Material Input Line Entry Specification; GEAM: Group Environment Annotation Marker; InChI: IUPAC International Chemical Identifier; MDAM: Molecular Detail Annotation Marker; MIAM: Miscellaneous Interest Annotation Marker; OPAM: OPerational Annotation Marker; SFN: Stoichiometric Formula Notation; SMILES: Simplified Molecular Input Line Entry System; SQC: SQuare bracket atomic Code; SSAM: State and Shape Annotation Marker

## Competing interests

The author declares that they have no competing interests.

## Authors' contributions

AD designed the CurlySMILES language. AD has implemented software to parse and test CurlySMILES notation and to use them in CurlySMILES-annotated archives of chemical property data and bibliographic collections.

## Supplementary Material

Additional file 1 **CurlySMILES encoding examples**. The encoding examples illustrate the application of CurlySMILES formats and rules to derive linear notations for selected structures including stereoisomers, fragments, ring molecules with delocalized charge, coordination compounds, macromolecules, nanostructures as well as doped and surface-functionalized materials. Click here for file
