# Supplementary material for: CurlySMILES: a chemical language to customize and annotate encodings of molecular and nanodevice structures
Source: J Cheminform. 2011 Jan 7;3:1. doi: 10.1186/1758-2946-3-1 (PMC3027187; doi:10.1186/1758-2946-3-1)
Supplement: Additional file 1 — CurlySMILES encoding examples. The encoding examples illustrate the application of CurlySMILES formats and rules to derive linear notations for selected structures including stereoisomers, fragments, ring molecules with delocalized charge, coordination compounds, macromolecules, nanostructures as well as doped and surface-functionalized materials. [file 1758-2946-3-1-S1.PDF]

CurlySMILES encoding examples

Axel Drefahl
Address: Axeleratio, Reno, Nevada, USA
Email: axeleratio@yahoo.com
Internet: http://www.axeleratio.com/csm/proj/main.htm

This document (Additional file 1) supplements the article entitled "CurlySMILES: a chemical language to customize and annotate encodings of molecular and nanodevice structures." The following examples (E1 to E20) illustrate the application of the CurlySMILES annotation grammar to encode chemical structures in various contexts.

E1: enantiomers. Enantiomers that result from the presence of one or more stereogenic (formerly: asymmetric) centers are specified by stereodescriptive annotation using symbols `D`, `L`, `R`, and `S` when a center coincidences with an atomic node. Respective annotations are anchored at the ANC, for example:

`O=C(O)C{R}(O)C{R}(O)C(=O)O`	dextrotartaric acid
`O=C(O)C{S}(O)C{S}(O)C(=O)O`	levotartaric acid
`O=C(O)C{R}(O)C{S}(O)C(=O)O`	mesotartaric acid

The historically significant D/L system relates chiral molecules to the D and L enantiomers of glyceraldehyde. Since it may occasionally still be convenient to assign these descriptors, they are included in the CurlySMILES language. The encoding examples for (*R*)-2,3-dihydroxypropanal (D-glyceraldehyde) and (*R*)-2-amino-3-sulfanylpropanoic acid (L-cysteine) demonstrate alternate use of these two stereodescription systems:

`O=CC{D}(O)CO` or `O=CC{R}(O)CO`	D-glyceraldehyde
`O=CC{L}(N)CS` or `O=CC{R}(N)CS`	L-cysteine

Molecules with more than one stereogenic center should always be encoded by using the `R` and `S` stereodescriptors, as shown for the glyceraldehyde derivative (2*R*,3*R*)-2,3,4-trihydroxybutanal, commonly known as D-erythrose:

`O=CC{R}(O)C{R}(O)CO`	D-erythrose

E2: *cis/trans*-isomers. Specification of *cis*/*trans* isomers that result from different substituent arrangement at a double-bond atom pair follows the rules of chemical nomenclature using the *E*/*Z* convention. The annotation is anchored at the ANC that follows the double-bond symbol in the notation. For example, the two stereoisomers of octadec-9-enoic acid are encoded as:

`O=C(O)CCCCCCCC=C{E}CCCCCCCC`	elaidic acid (*trans* form)
`O=C(O)CCCCCCCC=C{Z}CCCCCCCC`	oleic acid (*cis* form)

E3: groups. Structural unit annotation can result in uni- or multivalent substructures or groups. Examples for encoded univalent groups are:

`C{-}`	methyl group, H_3C-
`N{-}`	amino group, H_2N-
`O{-}`	hydroxy group, HO-
`F{-}`	fluoro group, F-

There is no limitation of group size:

`CO[Si]{-}(OC)OC`	trimethoxysilyl group, $(H_3CO)_3Si$-

Groups can have any number of structural unit annotations and a node can have a multiple of them:

`C{-}#CC#C{-}`	butadiyne unit
`C{#}C#CC{=}`	but-2-yne-1-yliden-4-ylidyne unit
`[c]1{-}c[c]{-}ccc1`	1,3-phenylene unit
`[Pt]{~}{~}{~}{~}`	tetravalent, coordinating platinum atom

E4: linked and embedded structures. Instead of simply encoding a group structure, a group can be encoded by including details of the structural environment. This is achieved with group environment annotations. The format is an extension of the structural unit annotation format:

`O{-R}`	hydroxy group in alkanols

Notice that this encodes a hydroxy group as a structural part of an alkanol, not the compound class of alkanols itself, which is encoded as `O{+R}` (see **E9**). More diverse environments are possible:

`[PH+]{-R}{-R}CC{-X}`	dialkyl-haloethyl-phosphonium cation

The environment can further be charactarized:

`C{-Rbra=0}(=O)O{-Rn=4-8}`	carboxylic group in alkyl *n*-alkanoate

This notation denotes a carboxylic group in an alkyl *n*-alkanoate. The key/value pair `bra=0` limits `R` to unbranched alkyl chains. The pair `n=4-8` defines an alkyl chain with 4 to 8 C-atoms, which can be both linear and branched (by default, `bra=1`). The key/value pair `bra=2` restricts `R` to branched-only groups. Again, to encode the corresponding set of alkyl *n*-alkanoates, the two `-R` markers need to be replaced by `+R`.
The site to which a group is attached can be described in detail by including a CurlySMILES notation of the site within the annotation, adding a dictionary entry with key `c`. The following notations represents a site-specific fluorine atom:

`F{-Yc=[c]{:}{:}{-}}`	flourine atom adjacent to an aromatic C atom

The site to which a group is attached can be described in detail by including a CurlySMILES notation of the site within the annotation, adding a dictionary entry with key `c`. The following notations represents a site-specific fluorine atom:

`F{-Yc=[c]{:}{:}{-}}` flour atom adjacent to an aromatic C atom

A proton at a platinum surface is represented as:

`[H+]{.|c=[Pt]}` H^+ at Pt surface

For comparsion, `[H][H]{ADc=[Pt]}` uses a MIAM-based annotation to represents a hydrogen molecule at a platinum surface.

E5: functionalized material surfaces. A functionalized material surface is represented by encoding the functionalizing molecular structure in SMILES and using a group environment annotation to specify the surface attachment. For example, the structure of a phenyl-capped CdSe crystallite can be encoded as

`c1cccc[c]1{-|c={*CdSe}}` phenyl group covalently linked to CdSe surface

or more compact as:

`c1cccc[c]1{-|sfn=CdSe}` phenyl group covalently linked to CdSe surface

E6: delocalized charge. Charge delocalization is specified by applying a molecular detail annotation to the participating atoms. For example, a delocalized ring charge is encoded via marker `!r` and a dictionary entry with key e and assignment of the charge value:

`c1cccc1{!re=-}` cyclopentadienide ($C_5H_5^-$) anion (**1a**)

`c1cccccc1{!re=+}` tropylium ($C_7H_7^+$) cation (**1b**)

`c1ccccccc1{!re=-2}` cyclooctatetraenide ($C_8H_8^{2-}$) dianion (**1c**)

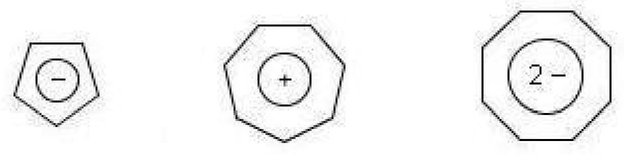

1a **1b** **1c**

E7: hydrogen-bonding. A hydrogen-bond is indicated with marker !H at the ANC of the hydrogen-donating atom. The node of the accepting atom is specified by assigning its index value to key `i`:

`O=C(O)c1ccccc1O{!Hi=1}` salicylic acid with intramolecular H-bond

Structures with intermolecular (inter-component) hydrogen-bonding require additional component specification, which is achieved by appending `#` followed by the component index

to the node index. For example, a cyclic dimer of two benzoic acid molecules can be encoded as:

```
c1ccccc1C(=O)O{!Hi=3#2}.O{!Hi=8#1}C(=O)c1ccccc1
```

The annotation `{!Hi=3#2}` at the hydroxy group in the first component indicates that the acceptor atom is the third atom in the second component. The annotation `{!Hi=8#1}` at the hydroxy group in the second component indicates that the acceptor atom is the eighth atom in the first component.

E8: ligand molecules. Site-specific non-hydrogen interactions are annotated with `!I`. Such annotation are used to indicate, for example, donor atoms in potential ligand molecules:

`N{!I}CCN`	ethylenediamine (en) as monodentate ligand
`N{!I}CCN{!I}`	ethylenediamine (en) as bidentate ligand

The molecule 2,2'-:6',2''-terpyridine (terpy) can be annotated as a tridentate ligand as follows:

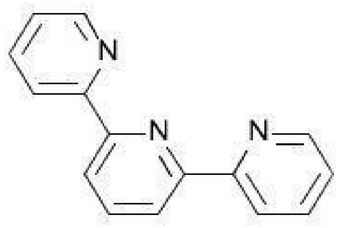

`n1{!I}ccccc1-c2n{!I}c(ccc2)-c3n{!I}cccc3`	2,2'-:6',2''-terpyridine

E9: molecule sets. A set of molecules is formally generated with annotations using markers `+R`, `+X`, and `+Y`. A set of positional isomers is generated by placing the position-changing substituent inside an annotation. The three xylene isomers, for example are encoded as toluene annotated with the position-changing methyl group:

`Cc1ccccc1{+Rc=C{-};p=4,5}`	the three xylene isomers

Annotation entry `p=4,5` refers to the *meta*- and *para*-positions, respectivley. Notice that p=7 (*ortho*-position) is not explicitly encoded since this position is determined by the anchor atom at which the position-defining annotation occurs.
Sets can virtually be infinite when a notation contains an annotation with a not further specified alkyl substituent an any-kind substituent:

`c1{+Xaa=Br,Cl}ccc{+R}cc1`	4-bromo- and 4-chloro-alkylbenzenes
`C1COCC[N+]1{+Rn=1,2}{+R}`	4-methyl- and 4-ethyl-4-alkylmorpholinium
`NN=C{+Y}{+Y}`	any hydrazone

E10: peptides and derivatives. An `+Y` annotation is also used to represent molecules with structural parts that can more efficiently be encoded by applying special schemes such as the three- or one-letter codes for proteinogenic amino acids. An unmodified peptide is encoded as a sequence of amino acids anchored to a hydrogen atom at its N-terminus. The tetrapeptide tuftsin, for example, is denoted as

`[H]{+Ypep=Thr-Lys-Pro-Arg}` H-Thr-Lys-Pro-Arg-OH

The three-letter symbols of the amino acids have to be separated by hyphens. A shorter notation is derived by using one-letter symbols without hyphen separation:

`[H]{+Ypep=TKPR}` H-TKPR-OH

A modified peptide or a complex molecule containing a peptide substructure is encoded by replacing [H] at the N-terminus by the notation for the non-peptide substructure. The OH group at the C-terminus, as shown above, is the default ending group and is not explicitly encoded. Any other group at the C-terminus is included into the sequence code by appending it as a CurlySMILES group notation, separated by an ampersand. Encoding of a modified peptide is demonstrated for the azidopeptide, in which the C-terminus ends with an amino group, $-NH_2$ encoded as `N{-}`[Structure from 10.1039/b507975f]:

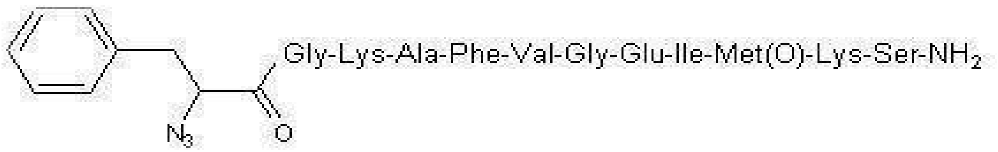


```
c1ccccc1CC(N=[N+]=[N-])C{+Ypep=GKAFVGEI-Met(O)-KS&N{-}}=O
```

Modified amino acids, such as methionine S-oxide (Met(O)), have to be inserted via hyphen separation, if a short notation is used. Instead, Met(O) can explicitly be encoded as CurlySMILES notation. It is then inserted into a sequence by enclosing it with ampersand characters. The above notation becomes

```
c1ccccc1CC(N=[N+]=[N-])C{+Ypep=\
    GKAFVGEI&N{-}C(CCS(=O)C)C(=O)O{-}&KS&N{-}(=O)
```

Such combined encoding of peptide chains and other structural parts, by formally inserting CurlySMILES-encoded substructures between peptide bonds or at the chain terminus, supports flexible encoding of various biomolecules while taking advantage of the universally accepted short notations for amino acids.

E11: assembled biomolecules. Large biomolecules are often formally presented by packaging certain structural parts into named entities such as abbreviated terms or acronyms. The lipase-BSA hetero-dimer is such a structure, in which the protein bovin serum albumin (BSA) is anchored onto the surface of the enzyme *Thermomyces Lanuginosa Lipase*) (TLL) by a click-chemistry derived linkage group:

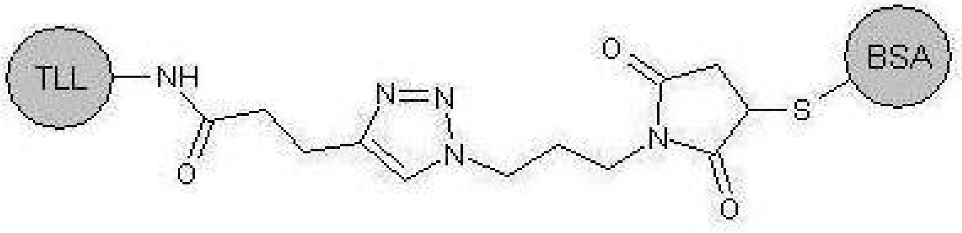

This structure [from 10.1039/b516551b] can be encoded as:

```
N{+Yenz=TLL}C(=O)CCc1nnn(c1)CCCN2C(=O)CC(C2=O)S{+Ypro=BSA}
```

The keys `enz` and `pro` are used to assign the enzyme and protein parts by their shorthand notation.

E12: homonuclear complexes. A homonuclear complex is represented in CurlySMILES by either listing the constituents via dot-separation or by annotating the nucleus with its ligands using the OPAM `+L`, as shown for the hexamminecobalt(III) cation:

`[Co+3].N{!Ii=1#1}{6}`	$[Co(NH_3)_6]^{3+}$, dot-based notation
`[Co+3]{+Lc=N{6}}`	$[Co(NH_3)_6]^{3+}$, OPAM-based notation

In the first notation, coordination entity and ligands occur at the same hierarchical level, whereas the second notation clearly separates the central cobalt ion from the amine ligands, which are formally coordinated (and subordinated) via OPAM-based annotation. An OPAM-based notation has the advantage that it explicitly declares a coordination molecule. Annotation of ligands with `{!I}` is optional, but critical to distinguish between different coordination possibilities in complexes with multiple donor atoms, as in the following Pt complex with a tridentate ligand:

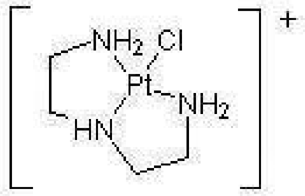


```
[Pt+2]{+Lc=[Cl-].N{!I}CCN{!I}CCN{!I}}
```

E13: repetition of a structural unit
Repetition of a structural unit is encoded with OPAM `+n`, formally repeating the unit to build a chain molecules. The notation for [bis[2-(2-methoxyethoxy) ethyl] ether demonstrates encoding of a molecule with a defined number of repetitions:

`CO{-}CC{+nn=4}OC`	$CH_3(\mathbf{OCH_2CH_2})_4OCH_3$

The repeated unit is represented in boldface. It is denoted as a bivalent group, while the second structural unit annotation of an open single bond is replaced by the `+n` marked annotation. The entry `n=4` specifies the number of repetitions. Macromolecules are encoded in the same way, but the number-entry is omitted unless one wants to specify a particular chain length. Poly[bis(methoxy-ethoxy-ethoxy)phosphazene] (MEEP) has the following notation:

`N{-}=P{+n}(OCCOCCOC)(OCCOCCOC)` MEEP

Since the two ether groups occur on the right side of the +n annotated phosphorus atom, they have to be enclosed in parenthesis to be both considered part of the repeated unit. Any notational part to the right of an +n annotated ANC, which is not indicated as a branch, is interpreted as externally continuing or as a terminal group. In contrast, in macrocylce notations using `+r` all parts of the notation are considered to be part of the repeated unit.

E14: rings build from a repeating unit

A ring molecule based on a repeating unit can be encoded by using OPAM `+r`. For example, the crown ether 18-crown-6 is encoded as

`C{-}CO{+rn=6}` $(CH_2CH_2O)_6$

An +r annotation is an efficient shorthand when the repeating units grow in size such as in calixarenes (see europium-calix[4]arene complex in the article).

E15: dissolved species. A dissolved species is annotated with SSAM `ds`. The solvent can be specified as CurlySMILES notation with the annotation, for example:

`ClCCCCCCC{dsc=ClC(Cl)(Cl)Cl}` 1-chloroheptane dissolved in CCl_4

If dissolved in water (H_2O), the annotation `aq` can be used as a shorthand. However, if dissolved in isotopically labeled water, such as heavy water (D_2O), the annotation marker `ds` is required and the annotation is `{dsc=[2H]O[2H]}`.

E16: crystal phases and polymorphs. The crystalline state of an encoded material can be indicated with SSAM `cr`. The phase of allotropes and polymorphs can be specified by a dictionary entry with key `all`, `phn`, `psy` and `spg` for allotrope name, phase name, Pearson symbol and space group symbol (Hermann-Mauguin notation). Diamond can be encoded as

`[C]{crall=diamond}` or `[C]{crpsy=cF8}` or `[C]{crspg=Fd-3m}`

Polymorphs of titanium dioxide (TiO_2) can be specified by their common phase names:

`{*TiO2}{crphn=anatase}`, `{*TiO2}{crphn=brookite}` and
`{*TiO2}{crphn=rutile}`

Redundancy is accepted and may even be welcome as cross-validation aid:

```
{*TiO2}{crphn=rutile;psy=tP6;spg=P4_2/mnm}
```

These crystal-phase-specifying dictionary entries can also be combined with other SSAMs such as `sc` for single-crystal, `nc` for nanocrystal, particular nanoforms (see next section) or with `tf` to define the crystal structure of a thin film.

E17: nanostructures. The nanoscale structure of a material is indicated with SSAM `ns`. More specific annotations such as `nc`, `nd`, `np`, or `nw` for nanocrystal, nanodisk, nanoparticle or nanowire are available. A zinc sulfide nanowire with wurtzite structure is denoted as:

`{*ZnS}{nwphn=wurtzite;psy=hP4;spg=P6_3mc}` wurtzite ZnS nanowire

Nanostructure notations can occur inside annotations, for example, to specify a surfactant molecules at the interface of a nanostructure such as *n*-dodecanethiol in a derivatized gold nanoparticle:

`CCCCCCCCCCCCS{-|c=[Au]{np}}` gold nanoparticle as substrate

E18: thin films. A thin film is specified with SSAM `tf`. A `tf`-marked annotation may include entries that describe the substrate, such as an entry with key `srf` to denote the substrate surface. The value is a SFN followed by the Miller-indices-based notation of the surface plane. A silicon dioxide thin film on a Si(1 0 0) substrate is encoded as:

`{*SiO2}{tfsrf=Si(100)}` SiO_2 thin film on Si(1 0 0) substrate

E19: surface-adsorbed molecules. Molecules at a material surface are encoded with MIAM `AD`. For example, tetradeuterosilane adsobed to a Ni(100) surface has the notation

`[2H][Si]([2H])([2H])[2H]{ADsrf=Ni(100)}` SiD_4 on Ni(100)

An AD annotation is used when the mode of molecule/surface attachment is not given in detail. Otherwise, when a molecule is attached or grafted to a surface via particular atoms, annotations with marker `~|`, `-|` or `.|` provide encoding option with higher precision.

E20: doped materials. An impurity or dopant is indicated with an `IM`-marked annotation, for example:

`[Si]{nw}{IMc=[P+]}` P^+-doped Si nanowire

`[Na+].[AlH4-]{IMa=Ti}` or `[Na+].[AlH4-]{IMc=[Ti]}` Ti-doped $NaAlH_4$

`{*ZnS}{IMa=Cr}{nwphn=wurtzite;spg=P6_3mc}` Cr-doped wurtzite ZnS nanowire

Additional resources

- Annotation dictionary
- Annotated SMILES notations
- Stoichiometric Formula Notation (SFN)
- Space group notations
- More encoding examples
